# Protecting Lives: A Quality Improvement Project to Improve Adherence to Fitness to Drive Policy in Mental Health Setting

**DOI:** 10.1192/bjo.2025.10390

**Published:** 2025-06-20

**Authors:** Sajid Mahmood, Tamara Chithiramohan, Omotilewa Omotoso, Waqqas Khokhar

**Affiliations:** Leicestershire Partnership NHS Trust, Leicester, United Kingdom

## Abstract

**Aims:** Driving is a complex and rapidly evolving task that requires a high level of skill and the ability to simultaneously interact with both the vehicle and the external environment. Mental illness can impair these abilities, potentially compromising both the driver’s safety and the safety of others. While individuals with mental health conditions have a legal obligation to refrain from driving if their condition renders them unfit, healthcare professionals, including doctors, have a crucial role to play. They are responsible for advising patients on the potential impact of their condition on driving ability, their legal duty to inform the Driver and Vehicle Licensing Agency (DVLA), and, in certain circumstances, directly notifying the DVLA on the patient’s behalf. Unfortunately, the driving status of patients is often overlooked during both admission and inpatient stays. There is also a concerning lack of awareness among patients regarding their duty to inform the DVLA and potential driving restrictions.

To mitigate these risks, it is essential to gather relevant information about driving status on admission, during the inpatient stay, and, most importantly, to discuss this with patients and carers at the time of discharge planning. The aims and objectives of this project were to achieve a 100% rate of driving risk assessment for all patients admitted to inpatient settings and to ensure that 100% of service users receive information about DVLA guidance following a mental health illness.

**Methods:** This Quality Improvement (QI) project involved assessing baseline practices against the local fitness to drive policy of Leicestershire Partnership NHS Trust. Data was collected from ten inpatient wards (six general adult and four older age) for patients discharged in January 2023. Information was gathered on driving risk assessment at admission, driving status at admission, driving risk assessment during the inpatient stay, and advice on fitness to drive given at discharge. An educational training video was developed and shared with trust-wide clinicians via email in September 2024, followed by a reminder email two weeks later. Data was collected again for patients discharged in October 2024.

**Results:** In the first cycle (January 2023), 128 patients were included. Driving risk assessment was completed for 95% of patients at admission. Approximately 12% of patients were driving at admission. Driving risk assessment during the stay was conducted in only 11.7% of cases. Advice on fitness to drive was given in 12.5% of cases. Notably, a quarter of driving patients did not receive such advice at discharge.

In the second cycle (October 2024), 85 patients were included. Driving risk assessment remained at 94%, similar to baseline. However, the proportion of driving patients increased to 20%. Driving risk assessment during the stay improved to 19%, a 7% increase. Advice on fitness to drive at discharge increased to 21.2%, an 8% increase. Surprisingly, the percentage of driving patients receiving advice decreased from 75% to 53%.

**Conclusion:** While there has been some improvement in compliance with the fitness to drive policy, significant gaps remain. To address this, it is crucial to enhance awareness among healthcare professionals regarding their role in driving risk assessment and patient advice. The educational training video will be made available on the uLearn training portal. It is recommended to incorporate this training into the induction process for new employees. Regular monitoring (biannually) of practices related to the fitness to drive policy is essential to further evaluate service delivery.

